# Common AZFc structure may possess the optimal spermatogenesis efficiency relative to the rearranged structures mediated by non-allele homologous recombination

**DOI:** 10.1038/srep10551

**Published:** 2015-05-22

**Authors:** Bo Yang, Yong-yi Ma, Yun-qiang Liu, Lei Li, Dong Yang, Wen-ling Tu, Ying Shen, Qiang Dong, Yuan Yang

**Affiliations:** 1 Department of Urology, State Key Laboratory of Biotherapy, West China Hospital, Sichuan University, Chengdu 610041, China; 2 Department of Medical Genetics, State Key Laboratory of Biotherapy, West China Hospital, Sichuan University, Chengdu 610041, China; 3 Reproductive Medicine Centre, West China Second Hospital, Sichuan University, Chengdu 610041, China; 4 Reproductive Medicine Institute, Chengdu Women’s & Children’s Central Hospital, Chengdu 610031, China

## Abstract

The azoopsermia factor c (AZFc) region of human Y-chromosome is an essential genomic segment for spermatogenesis with frequent non-allele homologous recombination (NAHR). Recent case-control studies on the association of the NAHR-based AZFc structural mutations with spermatogenic failure produced inconsistent results. To more precisely evaluate their spermatogenesis effects, we investigated the correlation between the subdivided AZFc mutations and sperm production in 3,439 Han Chinese males. Our results showed that both partial AZFc deletion-only and primary duplication mutation presented a significant risk for decreased sperm production. Restoration of the reduced dosage of the AZFc content to the normal level had a milder effect, whereas an overdose of the AZFc content arising from multiple duplications of a partial AZFc-deleted structure produced a more serious consequence compared to the partial deletion-only mutation. Additionally, the AZFc-mutated structures with excessive NAHR-substrate showed a notably negative effect on spermatogenesis. These results suggest that the recurrent NAHR-based AZFc mutations may be associated with decreased spermatogenesis efficiency in present population. More significantly, our finding implies that the overdose of AZFc NAHR-substrate may produce an additional risk for the massive AZFbc deletions during the multi-stage division process of germ cells and thus impair the global spermatogenesis efficiency in the carriers.

Azoospermia factor c (AZFc) region in the male-specific region on the Y chromosome (MSY) hosts a number of spermatogenesis-related gene families. It has been confirmed via multiple findings that a complete AZFc deletion (b2/b4 deletion) leads to azoospermia or severe oligozoospermia in different ethnic and geographic populations^1^. This chromosomal region is of interest because it is composed of massive, nearly identical, repeated amplicons that harbour a wide variety of structural mutations due to frequent non-allele homologous recombination (NAHR)^2^,^3^. Recent population-based association studies suggest that several recurring partial AZFc deletions (b1/b3, b2/b3, and gr/gr deletions with or without secondary duplication) and primary AZFc duplications (without deletion and duplication only) may impair spermatogenesis^1^,^4^,^5^,^6^,^7^,^8^,^9^,^10^,^11^,^12^, further supporting the theory that structural mutations in the AZFc region play a significant role in modulating spermatogenic output. However, the association studies analysing the outcome of partial AZFc deletions and duplications in different ethnic and geographic populations demonstrate significant variance^6^,^9^,^10^,^13^,^14^,^15^.

In addition to differences in the inclusion criteria of the subjects, the proportion of azoospermic *vs* oligozoospermic patients, methodology, and sample sizes among the studies, it is possible that the effect of certain AZFc structural mutations on spermatogenesis is closely tied to ethnicity and the geographic characterisation of the population, potentially as a result of the Y-chromosome haplogroup (Y-hg) distribution, the presence of Y-hg-specific constitutive AZFc structural mutations or the susceptibility of the mutations to secondary rearrangements^5^,^7^,^9^,^11^,^16^. However, an unavoidable issue is that, until now, research on the association of the subdivided AZFc structural mutations with spermatogenesis efficiency has been insufficient due to both the number of studies that have been performed and their sample sizes^12^,^17^,^18^. When considering the irreplaceable role of the AZFc region in human spermatogenesis, further studies on this topic will be helpful for clarifying the contribution of AZFc structural mutations on spermatogenesis failure and investigating the potentially effective mechanism of the mutations.

In the present study, a detailed subtyping of AZFc structural mutations was performed using a complete experimental procedure including AZFc-STS (sequence tagged sites) detection, AZFc gene copy analysis, and dosage analysis of deleted in azoospermia (*DAZ*), chromodomain protein, Y chromosome, 1 (*CDY1*) and basic protein, Y chromosome, 2 (*BPY2*). These analyses were performed in males with AZFc deletions and primary duplications who were screened from among 3,439 unrelated Han Chinese males, including 891 patients with idiopathic azoospermia, 1,366 patients with idiopathic oligozoospermia, and 1,182 controls with normozoospermia. The correlations between the subdivided AZFc structural mutations and sperm production, including sperm concentration and total motile sperm count (TMC) were investigated to evaluate their influence on spermatogenesis efficiency.

## Results

### Characteristics of AZFc structural mutations

**Figure 1:**
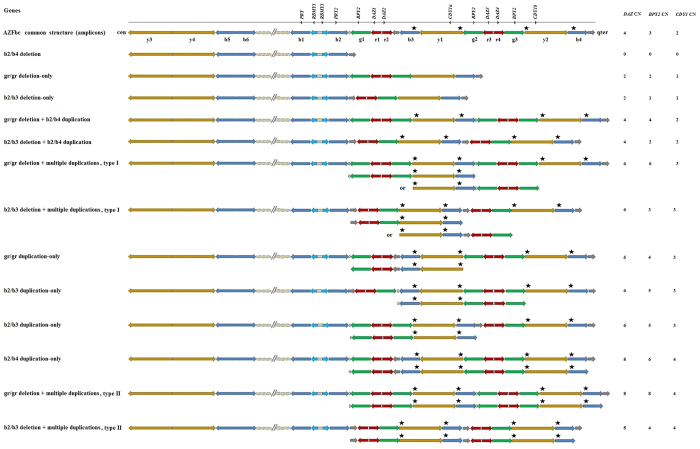
Schematic representation of the Y-chromosomal AZFbc region and the NAHR-based structural mutations. **** The location of AZFc genes and the term of AZFbc amplicons distinguished by different colours are shown in the common structure. The names of the mutations including AZFc deletions with or without secondary duplication(s) and primary AZFc duplications are shown on the left, and the copy dosage haplotypes of *DAZ*, *BPY2* and *CDY1* are shown on the right. The duplicated contents are shown below the integral AZFc structures. The total dosage of NAHR-substrate in the highly variable region of AZFc is consistent with the number of asterisks shown above the integral AZFc structures.

Upon completion of the analyses, 3 subgroups including 105 males with complete AZFc deletion, 327 males with partial AZFc deletion-only, and 158 males with partial AZFc deletion followed by secondary duplication(s) were screened from the 3,439 subjects collected between 2000 and 2014. Correspondingly, a total of 7 NAHR-based AZF deletion mutations were observed repeatedly in this study. The mutations included b2/b4 deletion, gr/gr and b2/b3 deletion-only, gr/gr and b2/b3 deletion + b2/b4 duplication, and gr/gr and b2/b3 deletion + multiple duplications (Fig. 1). They were superior in number in all of the observed deletion mutations (590/624, 94.6%). Among the deletions, only the b2/b4 deletion was found exclusively in the males with severe spermatogenic failure (sperm concentration ≤ 2.3 × 10^6^/ml).

The other 2 subgroups including 56 males with primary AZFc duplications mediated by NAHR and 696 males with common AZFc structure (reference AZFc sequence) were screened from the 913 out of 3,439 subjects collected between 2010 and 2014. Of note, 87.5% (49/56) of males with primary AZFc duplications were found to carry the 3 common dosage haplotypes of 6-4-3, 6-5-3 and 8-6-4 constituted by *DAZ*, *BPY2*, and *CDY1*, respectively. The haplotypes may stem from the occurrence of NAHR between the AZFc-amplicons, leading to the gr/gr, b2/b3 and b2/b4 duplication-only structures that were observed in this study (Fig. 1). There were no primary duplications observed exclusively in the males with spermatogenic failure; the gr/gr duplication-only was the most common mutation in the groups with different spermatogenic statuses.

When compared with the frequency of the partial AZFc deletion-only, the partial AZFc duplication-only was significantly lower (Fisher’s exact test, α = 0.05; 327/3,439, 9.5% *vs* 43/913, 4.7%, OR = 2.019, 95% CI =1.482-2.751, *P* < 0.001); however, after excluding the Q1 and N1 males with a constitutive partial AZFc deletion^4^,^7^,^9^, the frequency difference between the two AZFc mutations did not reach significance (Fisher’s exact test, α = 0.05; 177/3,439, 5.1% *vs* 43/913, 4.7%, OR = 1.093, 95% CI = 0.789-1.513, *P* = 0.330).

Moreover, we identified 34 males with atypical partial deletion + duplication mutation, whose dosage of AZFc genes could not be well-explained by NAHR of partial AZFc-deleted structure. Also, 7 males with atypical duplication-only mutation were observed, which showed an isolated dosage increase of *DAZ*, *BPY2* or *CDY1*. Totally, the distributions of the two mutations were similar between the patients with spermatogenesis failure and controls with normozoospermia.

### Correlation between the NAHR-based AZFc structural mutations and sperm production

First, we compared the proportion, the average age, the median sperm concentration and the total motile sperm count of the groups with different spermatogenic statuses between the total pool of 3,439 males and the 913 sampled males. In the comparisons, no significant difference was observed between the groups (Table S1), suggesting that the 913 males were a good representation of the population recruited during the period from 2000 to 2014 and that the sperm production was comparable among the males with the different AZFc structural mutations.

**Figure 2:**
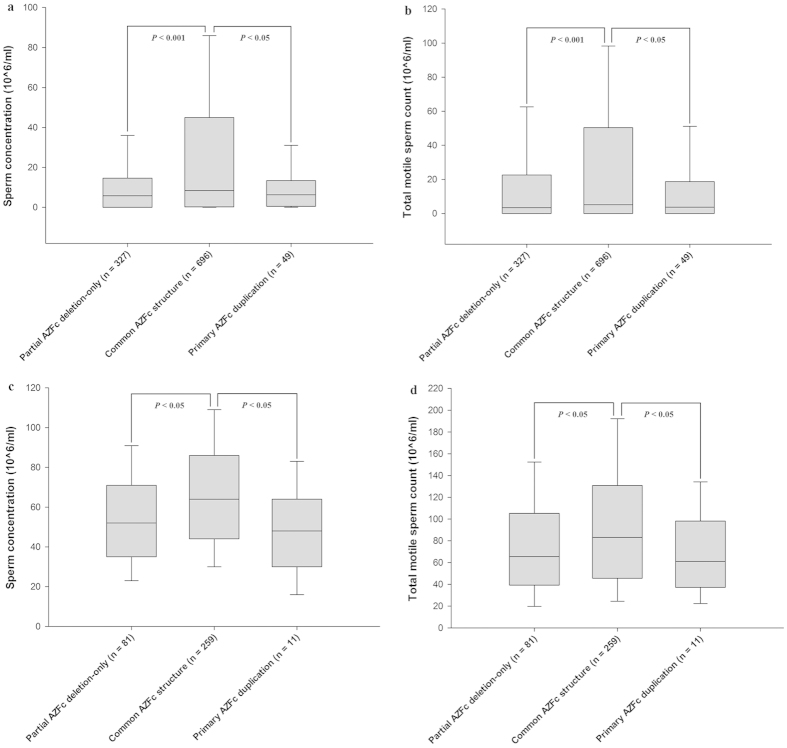
A comparison of sperm production between males with partial AZFc deletion-only or primary AZFc duplication and the common AZFc structure. **** The data are presented as the median with the 10th, 25th, 75th, and 90th percentile. (**a**) The males with partial AZFc deletion-only or primary AZFc duplication showed significantly lower sperm concentration relative to the common AZFc structure in groups without distinguishing spermatogenesis phenotype. (**b**) The males with partial AZFc deletion-only or primary AZFc duplication showed significantly lower TMC relative to the common AZFc structure in groups without distinguishing spermatogenesis phenotype. (**c**) The normozoospermic males with partial AZFc deletion-only or primary AZFc duplication showed significantly lower sperm concentration relative to the common AZFc structure. (**d**) The normozoospermic males with partial AZFc deletion-only or primary AZFc duplication showed significantly lower TMC relative to the common AZFc structure.

**Figure 3:**
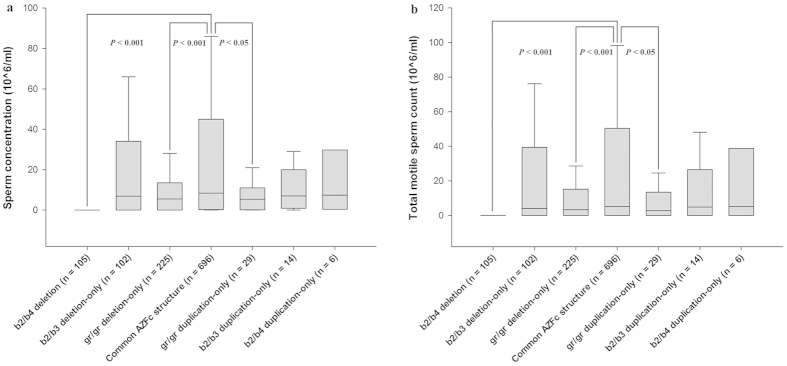
A comparison of sperm production between males with subdivided AZFc structural mutations and the common AZFc structure. **** The data are presented as the median with the 10th, 25th, 75th, and 90th percentile. (**a**) The males with b2/b4 deletion, gr/gr deletion-only, or gr/gr duplication-only showed significantly lower sperm concentration relative to the common AZFc structure in groups without distinguishing spermatogenesis phenotype. (**b**) The males with b2/b4 deletion, gr/gr deletion-only, or gr/gr duplication-only showed significantly lower TMC relative to the common AZFc structure in groups without distinguishing spermatogenesis phenotype.

Next, the median sperm concentration and total motile sperm count were compared between the males with common AZFc structure and mutated structures by reclassifying the groups based on the types of AZFc structural mutation, regardless of spermatogenesis status (Tables S2 and S3). To homogenise the AZFc structural background as much as possible, partial AZFc deletions with one or more secondary duplications were excluded from this analysis to avoid the potential interference of an overly complex structure. As a result, significant differences in sperm production were observed between males with common and mutated AZFc structures (Mann-Whitney *U* test, α = 0.05; sperm concentration: partial deletion-only, *P* < 0.001; primary duplication, *P *= 0.026; TMC: partial deletion-only, *P* < 0.001; primary duplication, *P* = 0.023) (Fig. 2a,b). As shown in Table S4, These comparisons in males with normozoospermia also showed similar results (Fig. 2c,d). More detailed comparison showed that males with gr/gr duplication-only, in addition to gr/gr deletion-only, presented a lower sperm production (Mann-Whitney *U* test, α = 0.05; sperm concentration: gr/gr deletion-only, *P* < 0.001; gr/gr duplication-only, *P* = 0.034; TMC: gr/gr deletion-only, *P* < 0.001; gr/gr duplication-only, *P* = 0.030) (Fig. 3). The findings strongly suggest that both partial AZFc deletion-only and primary AZFc duplication mutation significantly impair spermatogenesis efficiency.

**Figure 4:**
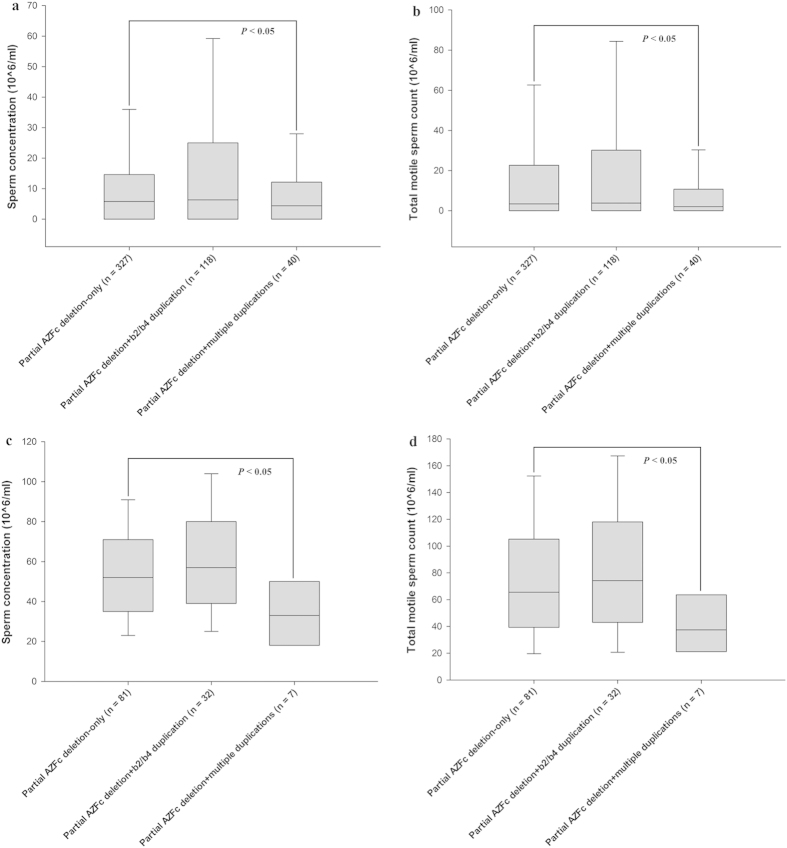
A comparison of sperm production between males with partial AZFc deletion followed by duplication(s) and partial AZFc deletion-only. **** The data are presented as the median with the 10th, 25th, 75th, and 90th percentile. (**a**) The males with partial AZFc deletion + multiple duplications showed significantly lower sperm concentration relative to the partial AZFc deletion-only in groups without distinguishing spermatogenesis phenotype. (**b**) The males with partial AZFc deletion + multiple duplications showed significantly lower TMC relative to the partial AZFc deletion-only in groups without distinguishing spermatogenesis phenotype. (**c**) The normozoospermic males with partial AZFc deletion + multiple duplications showed significantly lower sperm concentration relative to the partial AZFc deletion-only. (**d**) The normozoospermic males with partial AZFc deletion + multiple duplications showed significantly lower TMC relative to the partial AZFc deletion-only.

A sperm production analysis was performed for a partial AZFc deletion followed by one or more duplications. As shown in Fig. 4a,b, when compared with males harbouring the common AZFc structure, those with the partial deletion + multiple duplications showed much lower sperm concentrations, which was further confirmed in a comparison of the TMC (Mann-Whitney *U* test, α = 0.05; sperm concentration: *P* = 0.042; TMC, *P* = 0.035). However, males with the partial deletion + b2/b4 duplication had slightly increased sperm production relative to the partial deletion-only, although the difference was not significant (Table S5). The analysis was also performed in males with normozoospermia, and a similar result was found (Mann-Whitney *U* test, α = 0.05; sperm concentration: *P* = 0.035; TMC, *P* = 0.031) (Fig. 4c,d) (Table S6). The observations suggest that the b2/b4 duplication in a partial AZFc deletion structure may not produce more serious consequences, while the negative effect of the multiple duplications on spermatogenesis is more significant relative to a partial AZFc deletion-only.

### Correlation between the dosage of the AZFc NAHR-substrate sequence and sperm production

AZFc structural mutations lead to dosage alterations of AZFc amplicons, in addition to those of AZFc genes. This may produce an additional deletion load by increased dosage of the non-coding substrate sequence for NAHR and then confer a more significant risk for spermatogenic impairment in carriers relative to the common AZFc structure. If so, we expected that a correlation between the dosage of substrate sequence and sperm production would be observed.

**Figure 5:**
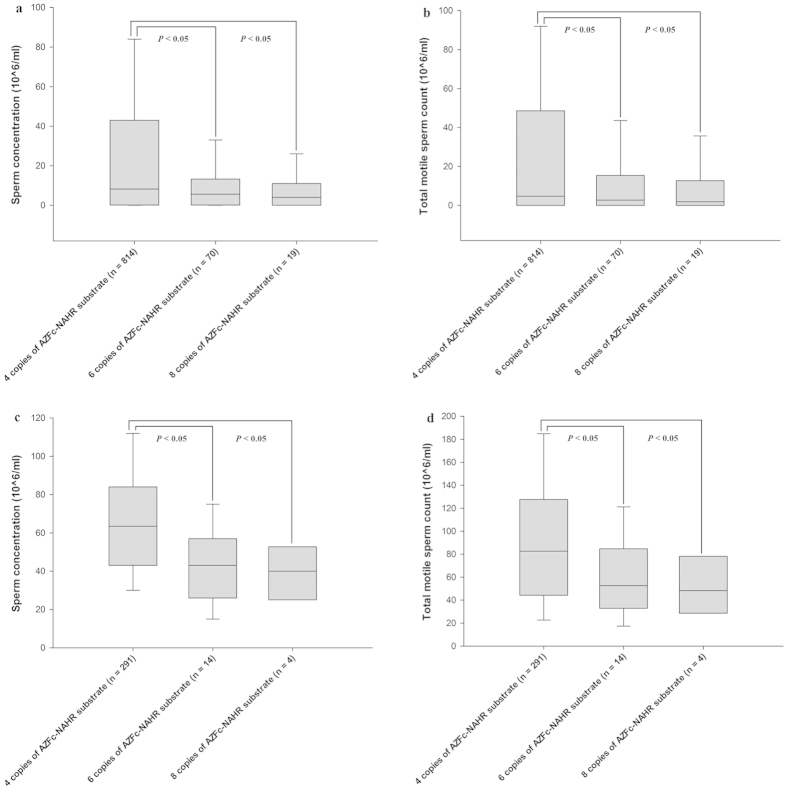
A comparison of sperm production between males with increased NAHR-substrate dosage and the common dosage. **** The data are presented as the median with the 10th, 25th, 75th, and 90th percentile. (**a**) The males with 6 or 8 copies of substrate sequence showed significantly lower sperm concentration relative to the common dosage in groups without distinguishing spermatogenesis phenotype. (**b**) The males with 6 or 8 copies of substrate sequence showed significantly lower TMC relative to the common dosage in groups without distinguishing spermatogenesis phenotype. (**c**) The normozoospermic males with 6 or 8 copies of substrate sequence showed significantly lower sperm concentration relative to the common dosage. (**d**) The males with 6 or 8 copies of substrate sequence showed significantly lower TMC relative to the common dosage.

To date, studies on massive deletions involved in AZFb, AZFbc or AZFc have identified two substrate sequences for NAHR resulting in these mutations, in which a 229 kb sequence is located in amplicon b and another 92 kb sequence is located in amplicon y^19^,^20^. In this case, we reclassified the three groups according to the total copy number of amplicons b and y in the 3’ region of b2 to b4. Group with 4 copies (2 copies of amplicon b and 2 copies of amplicon y) included males with the common AZFc structure and gr/gr and b2/b3 deletion + b2/b4 duplication; that with 6 copies included males with gr/gr and b2/b3 duplication-only and gr/gr and b2/b3 deletion + multiple duplications (type I in Fig. 1); that with 8 copies included males with a b2/b4 duplication-only and gr/gr and b2/b3 deletion + multiple duplications (type II in Fig. 1). Then, sperm production was compared between the group with 4 copies and that with 6 or 8 copies. The results showed that males with 6 or 8 copies of NAHR-substrate sequences presented a significantly lower sperm concentration and TMC relative to the group with 4 copies (Mann-Whitney *U* test, α = 0.05; sperm concentration: 6 copies, *P* = 0.012; 8 copies, *P* = 0.018; TMC: 6 copies, *P* = 0.010; 8 copies, *P* = 0.016) (Fig. 5a,b) (Table S7). This comparison was performed in males with normozoospermia, and a similar result was observed (Mann-Whitney *U* test, α = 0.05; sperm concentration: 6 copies, *P* < 0.001; 8 copies, *P* = 0.044; TMC: 6 copies, *P* < 0.001; 8 copies, *P* = 0.042) (Fig. 5c,d) (Table S8).

### Association of the NAHR-based AZFc structural mutations with spermatogenic failure

As shown in Table 1, the b2/b4 deletion was found exclusively in the patients with a significantly higher frequency relative to the controls. The deletion conferred a >4.7-fold increased risk for azoospermia relative to oligozoospermia (Fisher’s exact test, α = 0.05; 9.3% *vs* 1.6%, OR = 5.784, 95% CI = 3.643-9.184, *P* < 0.001). When combined with the presence of the b2/b4 deletion mutation in males with ≤2.3 × 10^6^/ml of sperm, the results indicate a b2/b4 deletion test for clinical purposes.

For the partial AZFc deletion-only mutation, a higher frequency in the patients with oligozoospermia or azoospermia was observed relative to the controls (Table 1). In more detail, a strong association of the gr/gr deletion-only on spermatogenesis failure was present, and such an effect of the b2/b3 deletion-only was not observed (Fisher’s exact test, α = 0.05; gr/gr deletion-only: 175/2,257, 7.8% *vs* 50/1,182, 4.2%, OR = 1.833, 95% CI = 1.349-2.491, *P* < 0.001; b2/b3 deletion-only: 71/2,257, 3.2% *vs* 31/1,182, 2.6%, OR = 1.199, 95% CI = 0.791-1.818, *P* = 0.227). Considering that Y-hg Q1 and N1 carry a constitutive gr/gr or b2/b3 deletion in the present population, we compared their spermatogenic effects with those of such mutations outside of the two Y-hgs. The results further confirmed that non-Y-hg Q1 males with partial AZFc deletion-only possessed a significant risk for impaired spermatogenesis (Fisher’s exact test, α = 0.05; gr/gr deletion-only outside of Y-hg Q1: 4.6% *vs* 2.7%, OR = 2.029, 95% CI = 1.385-2.974, *P* < 0.001; gr/gr deletion-only on Y-hg Q1: 2.0% *vs* 1.5%, OR = 1.484, 95% CI = 0.871-2.528, *P* = 0.089; b2/b3 deletion-only outside of Y-hg N1: 0.9% *vs* 0.3%, OR = 2.226, 95% CI = 0.751-6.600, *P* = 0.102; b2/b3 deletion-only on Y-hg N1: 2.4% *vs* 2.3%, OR = 1.047, 95% CI = 0.663-1.654, *P* = 0.473). Although the observations could not exclude the association of partial AZFc deletion-only on Y-hg Q1 and N1 with spermatogenesis failure, their spermatogenic effects could be much milder relative to such mutations outside of the two Y-hgs.

Furthermore, we investigated the influence of secondary duplication mutations in the partial AZFc-deleted structures on spermatogenesis. As shown in Table 1, a different distribution of the partial AZFc deletion + b2/b4 duplication mutation was observed between the controls and patients with oligozoospermia, implying that secondary duplication that restores the AZFc content to the normal level may still produce an unfavourable influence on spermatogenesis. However, the risk of males with partial AZFc deletion + b2/b4 duplication for azoospermia or oligozoospermia was relatively low with respect to those with partial AZFc deletion-only, although the association of the secondary duplication with azoospermia did not reach significance in this study. Of note, the distribution of the partial AZFc deletion + multiple duplications in patients and controls suggests that more duplication in the partial AZFc-deleted structure may confer a more significant risk for spermatogenesis failure in the carriers relative to those with partial AZFc deletion-only.

In relation to primary AZFc duplication mutations, an overall association with oligozoospermia was observed (Table 1), while only the contribution of the gr/gr duplication-only to oligozoospermia was identified in this study (Fisher’s exact test, α = 0.05; OR = 2.838, 95% CI = 1.059-7.604, *P* = 0.023). The results suggested an adverse effect of primary AZFc duplications on spermatogenesis.

## Discussion

Over the past 20 years, studies exploring the genetic causes of spermatogenic failure have produced many important achievements, in which the most remarkable finding has been Y-linked AZF microdeletions and their contribution to severe spermatogenic failure^1^,^19^,^20^. Among the 3 AZF regions, the AZFc region possesses the highest mutation rate resulting in a wide variety of NAHR-based structural deletions and duplications^3^. With the accumulation of data regarding the correlation of AZFc mutations to the spermatogenic phenotype, two issues have been clarified: the b2/b4 deletion, that is found exclusively in infertile males, is the known most common molecular cause for severe spermatogenic failure, and partial AZFc deletions confer a significant risk for impaired spermatogenesis^1^,^19^,^21^,^22^. However, except for b2/b4 deletion, the investigation into the influence of other recurrent AZFc structural mutations mediated by NAHR on spermatogenesis efficiency remains insufficient, and then it is unclear whether the mutations are of significance in the clinical workup of infertile couples.

Based on the copy dosage of *DAZ*, *BPY2* and *CDY1*, the present study revealed 4 NAHR-based AZFc deletion mutations (Table 1). In addition to the b2/b4 deletion presenting exclusively in males with severe spermatogenesis failure, 3 other mutations were more common in males with azoospermia and/or oligozoospermia relative to normozoospermic males. Totally, the 3 deletion mutations conferred a 1.6-fold risk for spermatogenesis failure in carriers with respect to those with the common AZFc sequence (Fisher’s exact test, α = 0.05; 365/2,257, 16.2% *vs* 120/1,182, 10.2%, OR = 1.593, 95% CI = 1.312-1.934, *P* < 0.001). Correspondingly, decreased sperm production for each mutation was identified in this study. The observations further suggest that not only the partial AZFc deletion-only but also such a deletion followed by duplication(s) may be adverse to spermatogenesis.

In more detail, in partial AZFc-deleted males, whether there is a compensatory role of the secondary b2/b4 duplication, which restores the decreased dosage of *DAZ* and *CDY1* to the normal level, to decreased spermatogenic efficiency of the partial deletion-only remains elusive. A multicentre study of European populations observed lower sperm production in males with gr/gr deletion + duplication(s) than those with gr/gr deletion-only^12^, while another study in the Dutch population reported that the secondary duplication restored the TMC to normal values in gr/gr-deleted men^18^. In this study with a larger sample size, slightly increased sperm production was found in the males with partial AZFc deletion + b2/b4 duplication with respect to those with partial AZFc deletion-only, implying that a secondary duplication may not lead to a higher risk for spermatogenesis failure via further impairing spermatogenesis efficiency. However, the males with partial AZFc deletion + multiple duplications presented a 1.6-fold risk for spermatogenesis failure with respect to the partial AZFc deletion-only (Fisher’s exact test, α = 0.05; partial AZFc deletion + multiple duplications, 33/2,257, 1.5% *vs* 7/1,182, 0.6%, OR = 2.469, 95% CI = 1.096-5.564, *P* = 0.015; partial AZFc deletion-only, 246/2,257, 10.9% *vs* 81/1,182, 6.9%, OR = 1.591, 95% CI = 1.250-2.024, *P* < 0.001). More importantly, a significant lower sperm production of men with partial AZFc deletion + multiple duplications was identified relative to partial AZFc deletion-only. The findings suggest that multiple duplications in the partially deleted AZFc region may produce a stronger negative effect on spermatogenesis than the other 2 subtypes of partial AZFc deletion mutations by more decreasing sperm production.

In this study, an overall association between the primary AZFc duplication mutation and oligozoospermia was confirmed, and for the first time, decreased sperm production correlated with the mutation. The results suggest that in addition to partial AZFc deletion, primary AZFc duplication may be another Y-linked risk factor for spermatogenesis failure by negatively influencing sperm production in the present population. Although only the effect of the gr/gr duplication was identified, that of the b2/b3 or b2/b4 duplication-only cannot be excluded before further investigation is performed in larger samples.

The essential role of the AZFc genes in spermatogenesis has been identified by the definite pathogenicity of the b2/b4 deletion, and the genes may retain expressed dosage-sensitive regulators^23^. In addition, the adverse effect of decreased AZFc gene dosage on sperm production has been suggested in recent reports^8^,^18^ and was confirmed in mixed or normozooperrmic subjects in the present study. However, it seems to be insufficient to explain the disturbance of subdivided structural mutations to spermatogenesis only with the dosage effect of AZFc genes. For example, why do males with partial AZFc deletion + b2/b4 duplication, presenting nearly the same dosage of AZFc genes as that of the common AZFc sequence, have lower spermatogenesis efficiency? Why does the excessive copy number of AZFc genes due to partial AZFc deletion + multiple duplications or primary duplication confer a significant risk for impaired spermatogenesis to carriers via dramatically decreasing sperm production? Therefore, aside from the gene dosage effect, we cannot determine whether there is a potential non-coding Y-linked structural factor that modulates the spermatogenic effect of the mutations. This speculation is likely to be reasonable. First, the DNA structural rearrangements may reflect a higher instability, as observed in an autosome- and X-chromosome-specific array-CGH study, which results in a more significant deletion load^24^. When the structural mutation presents in the AZFc region, the deletion load may lead to an increased proportion of AZFc-deleted germ cells in the carrier and thus cause a higher risk for spermatogenesis failure relative to the common structure. Second, a partial AZFc deletion had been suggested to be susceptible to complete AZFc deletion^5^,^25^, which implies that some AZFc structural mutations may possess significant risk for secondary deletion mutations due to the alteration of the free energy state of the rearranged AZFc region^13^. However, to date, the direct evidence for this is greatly limited, except for the report on the significant association of the copy number variation of *DYZ19* in AZFbc with spermatogenic failure^24^.

Here, our study on the subdivided AZFc structural mutations and their influence on sperm production may provide new evidence for the speculation above. When focusing on the AZFbc region, 4 known NAHR deletions cause severe spermatogenesis failure, including complete AZFc deletion (b2/b4 deletion) for severe oligozoospermia and azoospermia, complete AZFb deletion (P5/proximal P1 deletion) for azoospermia, and complete AZFbc deletion (P5/distal P1and P4/distal P1 deletion) for azoospermia^19^,^20^. The number of substrate sequences for homologous recombination may be one of the determining factors for the occurrence rate of recombination^26^. Of note, the AZFc structural mutations, especially the partial AZFc deletion + multiple duplications and primary AZFc duplications, increase the substrate dosage for massive AZFbc deletion in AZFc amplicons b and y (Fig. 1). This increase provides an opportunity to investigate the correlation between the substrate dosage and sperm production. We performed this analysis by reclassifying the groups based on the total dosage of amplicons b and y, and the results showed a decreasing tendency of sperm production with the increase of substrate dosage in the mixed or normozoospermic subjects. The finding offered novel evidence on the significant influence of non-coding AZFc structural factors on spermatogenesis. Different from the direct pathogenicity of a loss-of-function mutation, such as AZFc deletion-only, the alterations of the non-coding AZFc structure may impair their spermatogenic effect via more frequent AZFbc deletion rearrangements of sperm cells, which leads to an increased risk for spermatogenesis failure by impairing spermatogenesis efficiency, as a secondary pathogenesis. If it is the case, the observed association of partial AZFc deletion + multiple duplications or primary AZFc duplication with impaired spermatogenesis efficiency may be partially explained by the increased NAHR-substrate in the mutation structures.

In conclusion, this large-scale cohort study suggests that the recurrent AZFc structural mutations mediated by NAHR, including partial deletion-only, partial deletion followed by duplication(s), and primary duplication, may be adverse to spermatogenesis by decreasing spermatogenesis efficiency relative to the common AZFc structure in present population. The correlation between the increase of NAHR-substrate dosage and decreased sperm production implies that the non-coding AZFc structure may also be an important factor for AZFc mutation-related spermatogenesis failure, in addition to the copy dosage of AZFc genes. We speculate that this may partially account for the contribution of AZFc duplication mutation to impaired spermatogenesis. The novel findings strengthen our understanding to the reproductive effect of these mutations and will be significant in the clinical workup of infertile couples. Due to that the phenotypic consequence of AZFc structural mutations may be modified by other genome factors including the copy type of AZFc genes, gene conversions, and unknown structural variations of Y chromosome as well as environmental factors, the genotype-phenotype correlations should be further investigated in more populations.

## Methods

### Study population

A total of 3,439 unrelated Han Chinese males ranging from 21 to 43 years of age were recruited from the Sichuan Province in south-western China during a period from 2000 to 2014. The clinical data and peripheral blood samples were obtained from the Department of Medical Genetics and the Department of Urology, West China Hospital; the Reproductive Medicine Centre, West China Second Hospital, Sichuan University; and the Chengdu Reproductive Medicine Institute in Chengdu Women’s & Children’s Central Hospital. This study was approved by the Ethics Committee of Clinical Trials and Biomedical Research, West China Hospital, Sichuan University, and the methods were carried out in accordance with the approved guidelines. The informed consent was obtained from all of the study subjects.

The subjects consisted of 1,182 controls with normozoospermia (sperm count ≥ 15.0 × 10^6^/ml and a total sperm count ≥ 39.0 × 10^6^/ejaculate) and 2,257 patients with idiopathic spermatogenic failure, including 891 patients with azoospermia (no sperm in ejaculate after centrifugation) and 1,366 patients with oligozoospermia (sperm count < 15.0 × 10^6^/ml and a total sperm count < 39.0 × 10^6^/ejaculate). The total motile sperm count (TMC) was calculated by multiplying the total sperm count and the percentage of progressively motile spermatozoa. The mean values of the sperm parameters in the control group were as follows: sperm concentration, 65.1 ± 30.9 × 10^6^/ml; TMC, 94.0 ± 78.9 × 10^6^/ml; and % progressive motility, 43.9 ± 13.7. Those values in the oligozoospermic group were as follows: sperm concentration, 6.4 ± 4.3 × 10^6^/ml; TMC, 4.2 ± 4.8 × 10^6^/ml; and % progressive motility, 20.6 ± 18.6. All of the subjects underwent a minimum of two semen analyses that were defined according to the WHO criteria^27^. Patients with known causes of spermatogenic failure, such as obstruction or absence of the vas deferens, orchitis, cryptorchidism, varicocele, hypogonadotrophic hypogonadism or karyotype anomalies, were excluded. Patients with microdeletions involving the AZFa and AZFb regions were also excluded based on the results of a recent study^22^.

### Deletion screening of the AZFc-STSs

All of the subjects were screened for complete and partial AZFc deletions, including the b2/b4, gr/gr, b2/b3 and b1/b3 deletions, using AZF-STS markers in which the b2/b4 deletion was identified by the absence of sY254 and sY255, the gr/gr deletion was detected by the absence of sY1291 and sY1189, the b2/b3 deletion was detected by the absence of sY1191 and sY1192 and the b1/b3 deletion was detected by the absence of sY1197, sY1291 and sY1191^8^. For the PCR primers and conditions, please see GenBank accessions G38349, G65827, G72340, G73809, GF102061, G67168 and G67166.

### Gene dosage haplotyping of males with partial AZFc deletions

For the males with partial AZFc deletions, their copy numbers of *DAZ*, *BPY2* and *CDY1* were first quantified by multiplex paralogue ratio tests (PRTs)^28^,^29^. Briefly, a marker chosen from a select AZFc gene family was located and amplified from each copy, and simultaneously, the PCR primer for the marker also amplified another homologous locus with a known copy number outside of the AZFc region; this technique can be performed even if there is a several-base difference in the length between the two PCR products. Following amplification with fluorochrome (FAM)-labelled primers, the two PCR products were separated by capillary electrophoresis, and the copy number of the AZFc gene (paralogue ratio) was calculated by comparing the peak areas of the two PCR products. For each sample, the co-amplification of *DAZ⁄DAZL*, *CDY1⁄CDY2* and *BPY2*/*BPY2DP* was performed using 3-plex quantitative fluorescent (QF)-PCR. The PCR products were then separated on an ABI PRISM 3100 Avant Genetic Analyzer (Applied Biosystems, Foster City, CA, USA) (Figure S1). Detailed primer information is listed in Table S9.

Furthermore, the copy dosages of *DAZ*, *BPY2* and *CDY1* from the males with partial AZFc deletions were validated using TaqMan real-time qPCR and the ΔΔCt method. *ZFY* was used as the internal control gene in all of the tests. The primers and TaqMan probes were designed for the target sequences’ masked repeat regions and SNPs using AlleleID 7.0 (PREMIER Biosoft, USA). The specificities of the single and multiple PCR primer sets were confirmed with a BLAST search (http://blast.ncbi.nlm.nih.gov/) and with MFEprimer-2.0 (http://biocompute.bmi.ac.cn/CZlab/MFEprimer-2.0/)^30^. The efficiencies of the *ZFY* and the AZFc gene qPCRs were confirmed in advance using the standard curves of a 5-log dilution series of the targets. Due to the absence of samples with known copy numbers of *DAZ*, *BPY2* and *CDY1* that have been confirmed via either Southern blot or fluorescent *in situ* hybridisation, the actual copy number of the AZFc genes could not be determined by real-time qPCR. Instead, we confirmed the copy count by PRT via the detection of the dosage ratio. Briefly, in the qPCR runs, 3 samples with either the reference AZFc, the gr/gr deletion-only or the b2/b3 deletion-only sequence, as determined by PRT, were used as external calibrators (reference samples) for each batch. The copy dosage ratios of the 3 genes in the detected samples relative to those of the 3 reference samples were calculated to confirm the PRT results. To ensure the reliability of the results, the tests were performed three times per sample and the qPCR runs were excluded if the efficiency was <0.90 or >1.10, the *R*^*2*^ was <0.95 or a standard deviation of >0.2 was identified between the three replicates. Information regarding the primers and probes for the real-time qPCR are listed in Table S10. The gene dosages of the samples detected by the two methods were completely concordant.

### Gene copy analyses of *DAZ* and *CDY1*

For males suspected to carry the mutation of partial AZFc deletion + b2/b4 duplication based on the absence of STSs and the gene dosage haplotype, the copy type of *DAZ* and *CDY1* was analysed using restriction fragment length polymorphism (RFLP) analysis. The analysis is a useful tool to distinguish the above AZFc structural mutation from the reference sequence when considering the potential false negative deletion of AZFc-STSs in the present population^7^. sY587, a single nucleotide variant (SNV) locus in the *DAZ* gene family, was used to detect the deletion of *DAZ1*/*DAZ2* or *DAZ3*/*DAZ4*. Another SNV, located 7750 bp upstream of the 5’ end of the *CDY1* family, was used to distinguish the deletion of *CDY1a* or *CDY1b*. The PCR products of sY587 and *CDY1*-7750 were digested with the restriction enzymes *Dra*I and *Pvu*II. The digestion products were separated on a 2% agarose gel and visualized by ethidium bromide staining. The primer sequences, PCR and RFLP conditions were described previously^31^.

### Gene dosage haplotyping of males with primary AZFc duplications

From the 3,439 samples, we selected 913 males who were recruited during the period from 2010 to 2014. These samples included 308 males with normozoospermia, 236 with azoospermia and 369 with oligozoospermia. After excluding the samples with known complete and partial AZFc deletions, the remainder were used to perform copy number analyses of *DAZ*, *BPY2* and *CDY1* using PRT, followed by real-time qPCR, as described above, to distinguish the primary AZFc duplications and the reference AZFc sequence. The gene dosages of the samples detected by the two methods were completely concordant.

### Y-chromosome haplogrouping of males with partial AZFc deletions

The Y-hgs Q1 and N1 contain a constitutive gr/gr or b2/b3 deletion^4^,^7^,^9^. The Y-hg typing was performed to distinguish the Q1 or N1 haplogroups from the other haplogroups. For this experiment, a total of 5 highly informative polymorphic loci from the Y chromosome were used to define the Q1 and N1 Y-hgs in males with partial AZFc deletions. Y-hg N1 was identified by the mutation of M89, M9 and LLY22g, and Y-hg Q1 was identified by the mutation of M89, M9, M45 and M120.

### Statistical analysis

The comparisons of sperm production, including the sperm concentration and total motile sperm count, were performed with a non-parametric median test, accounting for the fact that the sperm production was not evenly distributed among the subjects. The frequencies of the AZFc structural mutations were compared between the males with normozoospermia and those with azoospermia/oligozoospermia using Fisher’s exact test. The statistical analyses were performed using the statistical package SPSS 17.0 for Windows (Chicago, IL, USA). For the Mann-Whitney *U* tests and Fisher’s exact tests, values of *P* < 0.05 were considered to be statistically significant.

## Additional Information

**How to cite this article**: Yang, B. *et al.* Common AZFc structure may possess the optimal spermatogenesis efficiency relative to the rearranged structures mediated by non-allele homologous recombination. *Sci. Rep.* **5**, 10551; doi: 10.1038/srep10551 (2015).

## Supplementary Material

Supporting Information Supplementary Figures 1-6

## Figures and Tables

**Table 1:** Comparisons of the distributions of NAHR-based AZFc mutations between males with normozoospermia and spermatogenesis failure.

**AZFc structural mutation**	**Gene copy number**			**Distributions of AZFc structural mutations**			**Fisher’s exact test (α** = **0.05)**			
	***DAZ***	***BPY2***	***CDY1***	**NZ, n/total (%)**	**AZ, n/total (%)**	**OZ, n/total (%)**	***P*** **values**^	**OR (95% CI)**	***P*** **values**&	**OR (95% CI)**
b2/b4 deletion*	0	0	0	0/1,182 (0.0)	83/891 (9.3)	22/1,366 (1.6)	<0.001		<0.001	
Partial deletion-only*	2	1~2	1	81/1,182 (6.9)	87/891 (9.8)	159/1,366 (11.6)	0.010	1.425 (1.066-1.904)	<0.001	1.699 (1.315-2.194)
Partial deletion + b2/b4 duplication*	4	2~4	2	32/1,182 (2.7)	31/891 (3.5)	55/1,366 (4.0)	0.188	1.285 (0.790-2.090)	0.042	1.487 (0.969-2.283)
Partial deletion + multiple duplications*	6~8	3~8	3~4	7/1,182 (0.6)	12/891 (1.3)	21/1,366 (1.5)	0.061	2.274 (0.899-5.753)	0.017	2.596 (1.107-6.085)
**Primary duplication**#	6~8	4~6	3~4	11/308 (3.6)	11/236 (4.7)	27/369 (7.3)	0.335	1.305 (0.576-2.958)	0.027	2.022 (1.020-4.009)

NZ, normozoospermia; AZ, azoospermia; OZ, oligozoospermia. Partial AZFc deletion-only included gr/gr and b2/b3 deletion-only. Primary AZFc duplication included gr/gr, b2/b3, and b2/b4 duplication-only.

^*^The data was obtained from 3,439 males recruited during 2000~2014.

^#^The data was obtained from the subgroup of 913 males recruited during 2010~2014.

^^^The comparisons of the frequencies of AZFc structural mutations between males with azoospermia and normozoospermia.

^&^The comparisons of the frequencies of AZFc structural mutations between males with oligozoospermia and normozoospermia.
