# Supplementary material for: Common AZFc structure may possess the optimal spermatogenesis efficiency relative to the rearranged structures mediated by non-allele homologous recombination
Source: Sci Rep. 2015 May 22;5:10551. doi: 10.1038/srep10551 (PMC4441132; doi:10.1038/srep10551)
Supplement: Supporting Information — Supplementary Figures 1-6 [file srep10551-s1.doc]

**Common AZFc structure may possess the optimal spermatogenesis efficiency relative to the rearranged structures mediated by non-allele homologous recombination**

**Bo Yang^1*^, Yong-yi Ma^2*^, Yun-qiang Liu^2^, Lei Li^3^, Dong Yang^4^, Wen-ling Tu^2^, Ying Shen^2^, Qiang Dong^1^ & Yuan Yang^2^**

^1^Department of Urology, ^2^Department of Medical Genetics, State Key Laboratory of Biotherapy, West China Hospital, Sichuan University, Chengdu 610041, China, ^3^Reproductive Medicine Centre, West China Second Hospital, Sichuan University, Chengdu 610041, China, ^4^Reproductive Medicine Institute, Chengdu Women's & Children's Central Hospital, Chengdu 610031, China.

Correspondence and requests for materials should be addressed to Y.Y. (yuan70323@263.net) or Q.D. (dqiang418@163.com)

^*^Both authors contributed equally to this work.

**Table S1∣The comparisons of the proportion, the average age and the sperm production between the males recruited during 2000~2014 and 2010~**2014

| Spermatogenesis status |  | Total population (n = 3,439) (2000~2014) | | | |  | Subgroup (n = 913) (2010~2014) | | | |
| --- | --- | --- | --- | --- | --- | --- | --- | --- | --- | --- |
|  |  | N (%) | The average age, mean ± SD | Median of SC (25th-75th) | Median of TMC (25th-75th) |  | N (%) | The average age, mean ± SD | Median of SC (25th-75th) | Median of TMC (25th-75th) |
| Normozoospermia |  | 1,182 (34.4) | 31.8 ± 4.8 | 62.0 (40.0-82.0) | 79.1 (43.4-126.3) |  | 308 (33.7) | 31.7± 4.6 | 61.0 (39.0-81.0) | 78.6 (42.6-124.5) |
| Azoospermia |  | 891 (25.9) | 30.2 ± 5.4 | 0 | 0 |  | 236 (25.8) | 30.5 ± 5.1 | 0 | 0 |
| Oligozoospermia |  | 1,366 (39.7) | 31.0± 5.0 | 6.2 (2.4-9.8) | 2.6 (0-7.1) |  | 369 (40.4) | 31.3± 4.8 | 6.1 (2.6-9.6) | 2.7 (0-7.4) |
| *P* value (α = 0.05) |  |  |  |  |  |  | 0.917^$^ | > 0.05 each^*^ | > 0.05 each^#^ | > 0.05 each^#^ |

SC, sperm concentration (n × 10^6^/ml); TMC, total motile sperm count (n × 10^6^/ml).

^$^Fisher’s exact test, α = 0.05; the comparison of the proportion between males recruited during 2000~2014 and 2010~2014.

^*^*t* test, α = 0.05; the comparison of the average age between males with the same spermatogenesis status recruited during 2000~2014 and 2010~2014.

^#^Mann-Whitney *U* test, α = 0.05; the comparison of median sperm production between males recruited during 2000~2014 and 2010~2014.

**Table S2**∣**The classifying information of the different AZFc structures (without secondary rearrangements)**

|  |  | Common AZFc structure^#^ |  | AZFc-deleted structures^*^ | | |  | Primary AZFc-duplicated structures^#^ | | |
| --- | --- | --- | --- | --- | --- | --- | --- | --- | --- | --- |
|  |  |  |  | b2/b4 deletion | gr/gr del-only | b2/b3 del-only |  | gr/gr dup-only | b2/b3 dup-only | b2/b4 dup-only |
| NZ, n/total (%) |  | 259/308 (84.1) |  | 0/1,182 (0.0) | 50/1,182 (4.2) | 31/1,182 (2.6) |  | 5/308 (1.6) | 4/308 (1.3) | 2/308 (0.6) |
| AZ, n/total (%) |  | 168/236 (71.2) |  | 83/891 (9.3) | 59/891 (6.6) | 28/891 (3.1) |  | 7/236 (3.0) | 3/236 (1.3) | 1/236 (0.4) |
| OZ, n/total (%) |  | 269/369 (72.9) |  | 22/1,366 (1.6) | 116/1,366 (8.5) | 43/1,366 (3.2) |  | 17/369 (4.6) | 7/369 (1.9) | 3/369 (0.8) |
| Total, n/total (%) |  | 696/913 (76.2) |  | 105/3,439 (3.1) | 225/3,439 (6.5) | 102/3,439 (3.0) |  | 29/913 (3.2) | 14/913 (1.5) | 6/913 (0.7) |

NZ, normozoospermia; AZ, azoospermia; OZ, oligozoospermia; del-only, deletion-only; dup-only, duplication-only.

^*^The data was obtained from 3,439 males recruited during 2000~2014.

^#^The data was obtained from the subgroup of 913 males recruited during 2010~2014.

**Table S3**∣**Comparisons of the median sperm productions between males with common and mutated AZFc structures**

| AZFc structures |  | Total, n |  | TCN |  | Median of SC (25th-75th) (n × 10^6^/ml) | *P* values^$^ |  | Median of TMC (25th-75th) (n × 10^6^/ml) | *P* values^$^ |
| --- | --- | --- | --- | --- | --- | --- | --- | --- | --- | --- |
| Common structure^#^ |  | 696 |  | 9 |  | 8.4 (0.2- 45.0) |  |  | 5.1 (0-50.4) |  |
| b2/b4 deletion^*^ |  | 105 |  | 0 |  | 0 | < 0.001 |  | 0 | < 0.001 |
| Partial deletion-only^*^ |  | 327 |  | 4~5 |  | 5.8 (0-14.6) | < 0.001 |  | 3.4 (0-22.6) | < 0.001 |
| b2/b3 deletion-only |  | 102 |  | 4 |  | 6.9 (0-34.0) | 0.108 |  | 4.1 (0-39.4) | 0.097 |
| gr/gr deletion-only |  | 225 |  | 5 |  | 5.5 (0-13.5) | < 0.001 |  | 3.3 (0-15.2) | < 0.001 |
| Primary duplication^#^ |  | 49 |  | 13~18 |  | 6.3 (0.5-13.4) | 0.026 |  | 3.7 (0-18.7) | 0.023 |
| gr/gr duplication-only |  | 29 |  | 13 |  | 5.4 (0.1-11.0) | 0.034 |  | 2.8 (0-13.4) | 0.030 |
| b2/b3 duplication-only |  | 14 |  | 14 |  | 7.0 (1.2-17.0) | 0.336 |  | 4.9 (0-26.5) | 0.341 |
| b2/b4 duplication-only |  | 6 |  | 18 |  | 7.4 (0.5-29.0) | 0.372 |  | 5.1 (0-37.6) | 0.398 |

SC, sperm concentration; TMC, total motile sperm count; TCN, total copy number of *DAZ*, *CDY1*, and *BPY2*.

^*^The data was obtained from 3,439 males recruited during 2000~2014.

^#^The data was obtained from the subgroup of 913 males recruited during 2010~2014.

^$^Mann-Whitney *U* test, α = 0.05; the comparisons between the groups with common and mutated AZFc structures.

**Table S4**∣**Comparisons of the median sperm productions between normozoospermic males with common and mutated AZFc structures**

| AZFc structures |  | TCN |  | Medians of SC (25th-75th) (n × 10^6^/ml) | *P* values^$^ |  | Medians of TMC (25th-75th) (n × 10^6^/ml) | *P* values^$^ |
| --- | --- | --- | --- | --- | --- | --- | --- | --- |
| Common structure (n = 259)^#^ |  | 9 |  | 64.0 (44.0-86.0) |  |  | 83.1 (45.6-130.9) |  |
| Partial deletion-only (n = 81) ^*^ |  | 4~5 |  | 52.0 (35.0-71.0) | 0.029 |  | 65.5 (39.3-105.2) | 0.020 |
| Primary duplication (n = 11) ^#^ |  | 13~18 |  | 48.0 (30.0-64.0) | 0.041 |  | 61.1 (37.2-98.1) | 0.039 |

SC, sperm concentration; TMC, total motile sperm count; TCN, total copy number of *DAZ*, *CDY1*, and *BPY2*. Partial AZFc deletion-only included gr/gr and b2/b3 deletion-only. Primary AZFc duplication included gr/gr, b2/b3, and b2/b4 duplication-only.

^*^The data was obtained from 1,182 normozoospermic males recruited during 2000~2014.

^#^The data was obtained from 308 normozoospermic males recruited during 2010~2014.

^$^Mann-Whitney *U* test, α = 0.05, the comparisons between the groups with common and mutated AZFc structures.

**Table S5∣Comparisons of the median sperm productions between males with partial AZFc deletion-only and such deletion followed by duplication(s)**

| AZFc structures |  | Total^*^, n |  | TCN |  | Median of SC (25th-75th) (n × 10^6^/ml) | *P* values^#^ |  | Median of TMC (25th-75th) (n × 10^6^/ml) | *P* values^#^ |
| --- | --- | --- | --- | --- | --- | --- | --- | --- | --- | --- |
| Partial deletion-only |  | 327 |  | 4~5 |  | 5.8 (0-14.6) |  |  | 3.4 (0-22.6) |  |
| Partial deletion + b2/b4 duplication |  | 118 |  | 8~10 |  | 6.3 (0-25.0) | 0.242 |  | 3.8 (0-30.1) | 0.235 |
| Partial deletion + multiple duplications |  | 40 |  | 12~20 |  | 4.4 (0-12.1) | 0.042 |  | 2.0 (0-10.7) | 0.035 |

SC, sperm concentration; TMC, total motile sperm count; TCN, total copy number of *DAZ*, *CDY1*, and *BPY2*. The partial AZFc deletion included b2/b3 and gr/gr deletion.

^*^The data was obtained from 3,439 males recruited during 2000~2014.

^#^ Mann-Whitney *U* test, α = 0.05; the comparisons between the groups with partial AZFc deletion-only and such deletion followed by duplication(s).

**Table S6**∣**Comparisons of the median sperm productions between normozoospermic males with partial AZFc deletion-only and such deletion followed by duplication(s)**

| AZFc structures |  | TCN |  | Medians of SC (25th-75th) (n × 10^6^/ml) | *P* values^$^ |  | Medians of TMC (25th-75th) (n × 10^6^/ml) | *P* values^$^ |
| --- | --- | --- | --- | --- | --- | --- | --- | --- |
| Partial deletion-only (n = 81)^*^ |  | 4~5 |  | 52.0 (35.0-71.0) |  |  | 65.5 (39.3-105.2) |  |
| Partial deletion + b2/b4 duplication (n = 32) ^*^ |  | 8~10 |  | 57.0 (39.0-80.0) | 0.182 |  | 74.2 (43.0-118.1) | 0.138 |
| Partial deletion + multiple duplications (n = 7) ^*^ |  | 12~20 |  | 33.0 (18.0-44.0) | 0.035 |  | 37.4 (21.2-63.6) | 0.031 |

SC, sperm concentration; TMC, total motile sperm count; TCN, total copy number of *DAZ*, *CDY1*, and *BPY2*. The partial AZFc deletion included b2/b3 and gr/gr deletion.

^*^The data was obtained from 1,182 normozoospermic males recruited during 2000~2014.

^$^Mann-Whitney *U* test, α = 0.05, the comparisons between the groups with partial AZFc deletion-only and such deletion followed by duplication(s).

**Table S7∣Comparisons of the median sperm productions between males with different dosage of AZFc NAHR-substrate sequence**

| Total dosage of AZFc NAHR-substrate |  | Total, n |  | Median of SC (25th-75th) (n × 10^6^/ml) | *P* values^ |  | Median of TMC (25th-75th) (n × 10^6^/ml) | *P* values^ |
| --- | --- | --- | --- | --- | --- | --- | --- | --- |
| 4 copies |  | 814**^*^** |  | 8.2 (0.1-43.0) |  |  | 4.7 (0-48.6) |  |
| 6 copies |  | 70**^#^** |  | 5.6 (0.1-13.3) | 0.012 |  | 2.7 (0-15.4) | 0.010 |
| 8 copies |  | 19**^&^** |  | 4.0 (0-11.0) | 0.018 |  | 1.9 (0-12.7) | 0.016 |

SC, sperm concentration; TMC, total motile sperm count.

^*^The subjects included 696 males with common AZFc structure and 118 males with partial deletion + b2/b4 duplication.

^#^The subjects included 29 males with gr/gr duplication-only, 14 males with b2/b3 duplication-only, 16 males with gr/gr deletion + multiple duplications showing the copy number haplotype of *DAZ*, *BPY2*, and *CDY1* as 6-6-3, and 11 males with b2/b3 deletion + multiple duplications showing that as 6-3-3.

^&^The subjects included 6 males with b2/b4 duplication-only, 9 males with gr/gr deletion + multiple duplications showing the copy number haplotype of *DAZ*, *BPY2*, and *CDY1* as 8-8-4, and 4 males with b2/b3 deletion + multiple duplications showing that as 8-4-4.

^Mann-Whitney *U* test, α = 0.05; the comparisons between the groups with 4 copies and more copies in AZFc region.

**Table S8∣Comparisons of the median sperm productions between normozoospermic males with different dosage of AZFc NAHR-substrate sequence**

| Total dosage of AZFc NAHR-substrate |  | Total, n |  | Medians of SC (25th-75th) (n × 10^6^/ml) | *P* values^ |  | Medians of TMC (25th-75th) (n × 10^6^/ml) | *P* values^ |
| --- | --- | --- | --- | --- | --- | --- | --- | --- |
| 4 copies |  | 291**^*^** |  | 63.5 (43.0-84.0) |  |  | 82.6 (44.3-127.6) |  |
| 6 copies |  | 14**^#^** |  | 43.0 (26.0-57.0) | < 0.001 |  | 52.6 (32.8-84.7) | < 0.001 |
| 8 copies |  | 4**^&^** |  | 40.0 (23.0-54.0) | 0.044 |  | 48.3 (28.2-79.0) | 0.042 |

SC, sperm concentration; TMC, total motile sperm count.

^*^The normozoospermic subjects included 259 males with AZFc reference structure and 32 males with partial deletion + b2/b4 duplication.

^#^The normozoospermic subjects included 5 males with gr/gr duplication-only, 4 males with b2/b3 duplication-only, 3 males with gr/gr deletion + multiple duplications showing the copy number haplotype of *DAZ*, *BPY2*, and *CDY1* as 6-6-3, and 2 males with b2/b3 deletion + multiple duplications showing that as 6-3-3.

^&^The normozoospermic subjects included 2 males with b2/b4 duplication-only and 2 males with gr/gr deletion + multiple duplications showing the copy number haplotype of *DAZ*, *BPY2*, and *CDY1* as 8-8-4.

^Mann-Whitney *U* test, α = 0.05; the comparisons between the groups with 4 target copies and more copies in AZFc region.

**Table S9**∣**Details of the primers used in QF-PCR of PRT**

| Primer name |  | Primer sequence (5′→3′) |  | Expected product size (locations of target loci) |  | Comment |
| --- | --- | --- | --- | --- | --- | --- |
| oMY953a |  | HEX-TATTGAGACCCTTGCACCTG |  | 141bp (*CDY1* in AZFc-amplicon y) |  | Reference 31 |
| o1023 |  | GTTTCTTGGAGTTTCCCTTCTGTCACC |  | 144bp (*CDY2* in AZFb) |  |
| RE10F |  | HEX-GTGTGAAGGGAGAAAAGGGG |  | 228bp (*DAZ* in AZFc-amplicon r) |  | Present study |
| RE10R |  | GTTTCTTACAGGGACCCAGAAACCTTT |  | 234bp (*DAZL* in 3p24) |  |
| GB16F |  | HEX-TGAGGTTGTGGGTTGCATAT |  | 265bp (*BPY2* in AZFc-amplicon g) |  | Present study |
| GB16R |  | GTTTCTTTGGCCAGATCCACATATGAAGG |  | 268bp (*BPY2DP* in Yp11) |  |

**Table S10∣Details of the primers used in TaqMan real-time qPCR**

| Gene |  | Primer and probe sequence (5′→3′) |  | Product size (bp) |
| --- | --- | --- | --- | --- |
| *ZFY* |  | Forward: GATCCTCTGACGAGTGACATA / Reverse: CTTGCTGGTCCACTGAGA  Probe: HEX-CCGCTGGCATCTATGACTGCTTCA-BHQ1 |  | 100 |
| *BPY2* |  | Forward: GCAGAATTTGGTGGTGTGA / Reverse: GCCCAAGTGACCTGTCTA  Probe: FAM-CACTCCTTCAACGCACAGCCTCA-BHQ1 |  | 105 |
| *DAZ* |  | Forward: GCCTCTGTTCCTCCTTGA / Reverse: CCCTCAGGTCTTTCCTTCA  Probe: FAM-CTGCTGCCTGCCACCACCAT-BHQ1 |  | 76 |
| *CDY1* |  | Forward: CAGGATATGATTCAGCCCATTG / Reverse: GAGTCGTAAACAGTAATCAGTATCC  Probe: FAM- AGTCCGTGTATCACATACTGGCATCTGT-BHQ1 |  | 111 |

**
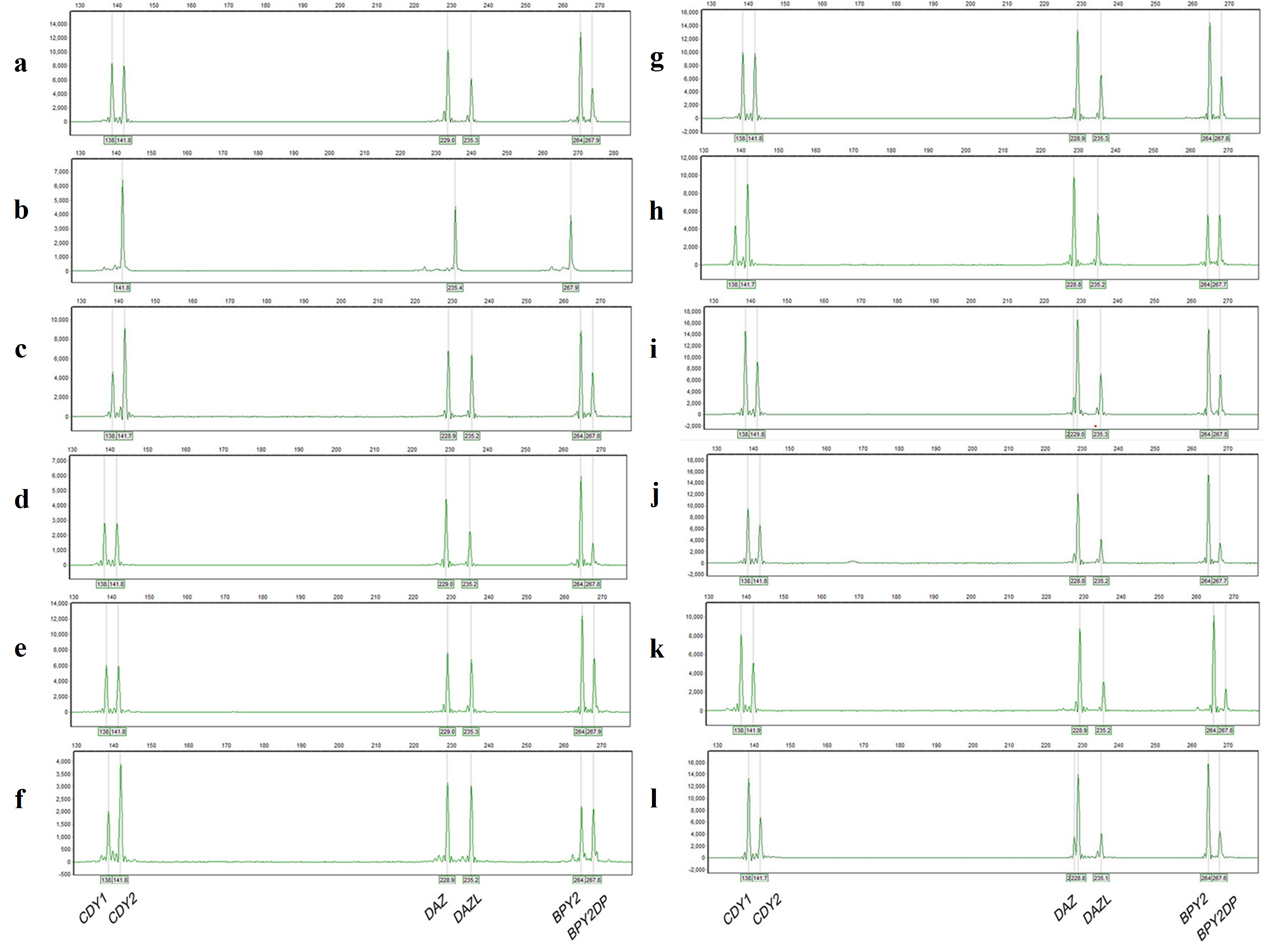
**

**Figure S1**∣**Examples of the AZFc structural mutations with the copy dosage haplotype of *CDY1*, *DAZ* and *BPY2* detected by PRT.** (a) The common AZFc structure with a copy dosage haplotype of 2-4-3. (b) The b2/b4 deletion with a copy dosage haplotype of 0-0-0. (c) The gr/gr deletion-only with a copy dosage haplotype of 1-2-2. (d) The gr/gr deletion + b2/b4 duplication with a copy dosage haplotype of 2-4-4. (e) The gr/gr deletion + *CDY1* duplication-only with a copy dosage haplotype of 2-2-2. (f) The b2/b3 deletion-only with a copy dosage haplotype of 1-2-1. (g) The b2/b3 deletion + b2/b4 duplication with a copy dosage haplotype of 2-4-2. (h) The b2/b3 deletion + *DAZ* duplication-only with a copy dosage haplotype of 1-4-1. (i) The b2/b3 deletion + multiple duplications with a copy dosage haplotype of 3-6-3. (j) The gr/gr duplication-only with a copy dosage haplotype of 3-6-4. (k) The b2/b3 duplication-only with a copy dosage haplotype of 3-6-5. (l) The b2/b4 duplication-only with a copy dosage haplotype of 4-8-6.
